# Evaluation of a Pharmacist–Dietician-Led Patient-Centered Approach to Managing CKD-MBD: A Mixed-Method Study

**DOI:** 10.3390/pharmacy8030171

**Published:** 2020-09-14

**Authors:** Tamara Baker, Heather Naylor, Bryanne MacNeil, Martin MacKinnon

**Affiliations:** 1 Department of Pharmacy, Nova Scotia Health Authority (Central Zone), QEII Health Sciences Centre, Halifax, NS B3K 4N1, Canada; bryanne.macneil@nshealth.ca; 2 Department of Pharmacy, Horizon Health Network, Saint John Regional Hospital, Saint John, NB E2L 4L4, Canada; heather.naylor@horizonnb.ca; 3 Department of Internal Medicine, Horizon Health Network, Saint John Regional Hospital, Saint John, NB E2L 4L4, Canada; martin.mackinnon@horizonnb.ca

**Keywords:** chronic kidney disease, focus groups, mineral and bone disorder, pharmacist, dietician, medication, patient-centered care, pharmacist prescribing, end-stage renal disease, dialysis

## Abstract

Chronic kidney disease mineral and bone disorder (CKD-MBD) is a common complication in end-stage renal disease (ESRD). To improve prescribing consistency and patient outcomes, a patient-centered, pharmacist–dietician-led approach to managing CKD-MBD was developed. The purpose of this study was to evaluate if the new approach impacted serum markers of CKD-MBD and medication burden, and to evaluate patient satisfaction. A single-arm, pre–post, mixed-methods study was conducted. Serum markers of CKD-MBD and medication data were collected pre- and post-intervention, and a patient survey administered post-intervention. Focus groups were conducted, transcribed, and analyzed thematically. No statistically significant differences in serum markers of CKD-MBD or medication burden were found. Eighty-seven percent of patients were satisfied with their care, however, 31% were very dissatisfied with medical explanations provided to them and 48% felt their allotted time with healthcare professionals was too short. Four major themes identified from focus groups included lack of privacy, knowledge and perceptions of blood work rounds, issues with taking phosphate binders, and areas for increased patient education. Patients would prefer more information regarding their blood work results and more time with the healthcare team. Areas for expanded education include renal diet, phosphate binders, and consequences of abnormal bloodwork.

## 1. Introduction

Disorders of bone and mineral metabolism are a common complication in patients with end-stage renal disease (ESRD). Chronic kidney disease mineral and bone disorder (CKD-MBD) can manifest as one or more of the following: abnormalities of calcium, phosphate, parathyroid hormone (PTH), or vitamin D metabolism; abnormalities in bone turnover, mineralization, volume, or strength; and vascular or other soft tissue calcification [1]. Hyperphosphatemia is a common complication of CKD-MBD and is a known risk factor for vascular calcification, cardiovascular morbidity, and mortality [2,3,4]. Treatment strategies for hyperphosphatemia in ESRD patients include removing phosphate via hemodialysis, restricting dietary intake of phosphate, and use of oral phosphate binders [5,6]. Hyperphosphatemia and CKD-MBD are often managed by a multidisciplinary healthcare team made up of nephrologists, nurses, pharmacists, and dietitians.

ESRD patients participating in focus groups have expressed a desire to be more involved in their care decisions [7,8]. In a thematic synthesis of qualitative research on patients undergoing in-center hemodialysis, patients expressed that they wanted more information from their healthcare providers and wanted to be listened to and involved in decisions about their care [7]. Discordance between the degree to which dialysis patients wish to be involved in their care and the degree of their actual involvement has also been noted in the literature [8]. Engaging patients through the implementation of patient-centered care has been associated with improved patient and provider satisfaction, improved patient outcomes, and reduced healthcare costs [9]. In addition, using a patient-centered approach has been shown to be especially relevant in medically complex patients, such as patients with chronic kidney disease, and in situations where there is no clear benefit to one treatment over another, as can be the case with various phosphate binders [10]. 

At our hemodialysis center, CKD-MBD in ESRD has traditionally been managed through monthly blood work rounds, led by a nephrologist and attended by a nurse, a renal pharmacist, and a renal dietitian. This approach has proven problematic in two respects: (1) there is minimal patient input in treatment choices, and (2) there is an inconsistent approach to the management of CKD-MBD due to varying prescribing practices among nephrologists. Therefore, a new integrative, patient-centered approach to monthly blood work rounds was developed in order to engage patients in their care and better standardize the approach to CKD-MBD management. With the new approach, patient goals and prognosis are used to determine serum phosphate targets and medication options. Furthermore, the new approach enables renal pharmacists to extend their traditional role in this area of practice by allowing them to prescribe and manage CKD-MBD-related medications through a collaborative practice. 

To our knowledge, no studies have evaluated a patient-centered approach to managing CKD-MBD led by pharmacists and dieticians. One small study done in chronic hemodialysis patients reported significant reductions in serum phosphate levels with a pharmacist–dietician-led protocol incorporating patient education, dietary counselling, and pharmacotherapy [10]. The protocol used in the study, however, did not stratify patients based on their goals and prognosis. The present study therefore adds to the very limited research that has been done in this area. 

The purpose of this study was to evaluate if there was a difference in serum markers of CKD-MBD (calcium, phosphorus, PTH) or medication burden post implementation of a pharmacist–dietitian-led, patient-centered approach to managing CKD-MBD in hemodialysis patients compared with the program’s previous standard of practice. The study also sought to describe patients’ experience, level of satisfaction, and degree of involvement with their care post implementation of the novel approach.

## 2. Materials and Methods

### 2.1. Study Design

An integrative, patient-centered approach to monthly blood work rounds was developed using the 2017 KDIGO (Kidney disease: improving global outcomes) CKD-MBD guidelines [11], and consensus based on clinical experience of the team members (Appendix A). Prior to developing the new approach, the program was following the 2009 KDIGO CKD-MBD guidelines [12]. A single-arm, pre–post, mixed-methods study was conducted at the Saint John Regional Hospital in Saint John, New Brunswick, Canada. This research study was reviewed and approved by the Saint John Regional Hospital Research Ethics Board on 27 November, 2017 (#2017-2532).

### 2.2. Participants

All adult patients (≥19 years of age) who had been receiving hemodialysis at the Saint John Regional Hospital for at least 4 months prior to implementation of the new approach to managing CKD-MBD were approached to consent to enrolment in the study. Patients were excluded if they had a diagnosis of dementia or were living in a nursing home.

### 2.3. Data Collection

#### 2.3.1. Quantitative

Each patient meeting inclusion criteria was assessed by the primary investigator using a specified data collection form and codebook. Baseline demographic and clinical characteristic data were collected, including age, sex, length of time on dialysis, transplant list status, and presence or absence of vascular calcification. Patients eligible for transplant were defined as being active on the transplant list, being worked up for transplant, or transplant status currently on hold. Patients were considered to have presence of vascular calcification if any X-ray completed in the previous 10 years had a note of vascular calcification in the description. Control data were collected retrospectively for the four months before implementation of the pharmacist- and dietician-led patient-centered approach. The experimental data was collected prospectively for five months after implementation of the new approach. Each patient served as his or her own control. Average serum phosphate, calcium, parathyroid hormone, number and class of CKD-MBD-related medications, and number of monthly medication changes were collected using the electronic health record pre and post intervention. Serum markers of CKD-MBD were measured monthly. At the end of the post-intervention period, a Short Assessment of Patient Satisfaction (SAPS) was distributed to each participant and responses were collected (Appendix B). The SAPS is a short, seven-item scale assessing the core domains of patient satisfaction which include treatment satisfaction, explanation of treatment results, clinician care, participation in medical decision-making, respect by the clinician, time with the clinician, and satisfaction with hospital/clinic care [13]. Responses are indicated on a five-point scale. Participants had the option to fill out the survey themselves or to have the investigator read the survey aloud and fill it out for them. All quantitative data were collected manually using a codebook designed by the primary investigator and recorded onto a password-protected Microsoft Excel spreadsheet. Data were analyzed using IBM SPSS version 22 statistical software.

#### 2.3.2. Qualitative

Participants were given the option to participate in a focus group regarding their experiences with and perspective of monthly blood work rounds. The primary investigator and a member of research services collected qualitative data through focus groups using a descriptive–exploratory method. Patients were asked to describe their experience with monthly blood work rounds and taking medications for CKD-MBD. A semi-structured interview guide was used to conduct focus groups and collect qualitative data from participants (Appendix C). 

### 2.4. Data Analysis

#### 2.4.1. Quantitative Analysis

All baseline characteristic were analyzed descriptively. To assess serum markers of CKD-MBD and medication burden pre and post intervention, the data were analyzed using a repeated measures analysis of variance by comparing these values at two time points, the end of the pre-intervention period and the end of the post-intervention period. Alpha of 0.05 was used to establish statistical significance. Effect size partial eta-squared (η_p_^2^) was reported for each analysis with values of 0.01, 0.06, and 0.14 reflecting small, medium and large effects, respectively [14]. The SAPS results were analyzed by frequency assessment of responses. 

#### 2.4.2. Qualitative Analysis 

Qualitative data analysis was done via content analysis. All taped focus groups were transcribed verbatim by the primary investigator and analyzed by members of the research team (TM, BM, HN) using thematic analysis. Researcher notes were analyzed for supporting data for the transcriptions. Transcripts were read and coded by three authors independently, who compared and discussed their individual coding choices and resolved any disagreements by discussion. Through a process of analysis and comparisons, categories of descriptive themes were inductively developed from the data.

## 3. Results

Eighty patients were enrolled in the study. Six patients did not meet inclusion criteria. Seventy-four patients consented to having their demographic, medication, and serum data collected. Of those 74 patients, 48 completed the SAPS survey. Additionally, 9 patients participated in focus groups. The mean age of participants was 67 years (SD = 14.4). Approximately 60% of participants were male, with an average length of time on dialysis of 4.8 years (SD = 3.2). An estimated 28% of participants were eligible for transplant, and 70% of patients had some evidence of vascular calcification (Table 1). 

### 3.1. Quantitative Results

There were no significant changes in serum calcium, phosphate, or PTH levels pre to post intervention (Table 2). There were no significant changes in the average number of medications or the average number of milligrams or tablets per day across time. There was a trend-level significance noted in average number of milligrams per day of calcium from the end of the pre-intervention period (M = 2191, SD = 1158) to the end of the post-intervention period (M = 2018, SD = 1125), *p* = 0.054, η_p_^2^ = 0.082. There was also a trend-level significance noted in average number of tablets per day of calcium from the pre-intervention period (M = 4.16, SD = 2.4) to the post-intervention period (M = 3.87, SD = 2.3), *p* = 0.074, η_p_^2^ = 0.071 (Table 3). 

Of the patients who participated in the SAPS survey, 87% had an overall survey result that indicated they were satisfied or very satisfied with their care. Approximately 96% of patients indicated they were satisfied or very satisfied with their choices in decisions affecting their healthcare. Patients felt respected by the healthcare professional all of time (73%) or most of the time (27%). When asked about explanations the team has given about results of their treatment or care, 56% of patients indicated they were satisfied or very satisfied, while 31% of patients indicated they were very dissatisfied. Additionally, 67% of patients surveyed either agreed or strongly agreed that the time they had with the doctor or other healthcare professional was too short. Further results from the SAPS can be viewed in greater detail in Table 4. 

### 3.2. Qualitative Results

Four major themes and four minor themes were identified from the two focus groups. The four major themes were titled lack of privacy, knowledge and perception of bloodwork rounds, issues with taking phosphate binders, and areas for increased patient education (Table 5). The four minor themes were titled living with chronic illness, satisfaction with care, individualized care, and fluid and dry weight (Table 6). 

#### 3.2.1. Major Themes

##### Lack of Privacy

Participants indicated that “nothing is confidential” in the dialysis unit and felt that the dialysis conditions did not allow for privacy when discussing their personal information with the healthcare team. Participants expressed a general discomfort with overhearing other patients’ information, and with having other patients overhear their information. They revealed that often they may not share things or ask any questions due to the lack of confidentiality on the unit. One participant said, “When the group is there, you may have a tendency to not say a whole lot of things you should be saying”. Another participant said, “So you can’t pose a question you might find embarrassing or questionable to somebody else, and you know basically you don’t want anyone knowing your business.” They stressed that this lack of privacy seemed somewhat contradictory to how privacy is usually prioritized in healthcare. One patient said, “They push confidentiality but then you’re in a big open pod”.

##### Knowledge and Perception of Bloodwork Rounds

A second major theme identified was patient’s knowledge and perceptions of blood work rounds. In general, patients felt that blood work rounds were a good process, keep patients informed, and are a helpful time for posing questions and having them answered. They perceive blood work rounds to be when healthcare professionals (including a doctor, a dietician, and a pharmacist) come to tell them about their bloodwork levels in the clinic. They did note that time spent with the healthcare team is too short and that time feels limited. One patient said, “If they come to see you it’s like okay, I’m gonna hit the top two biggest points and I’ll save the rest. I don’t want to tie them up because they’re going to see 30 people today. Another patient said, “You can’t really take the time. Nothing against the doctors but they’re in and they’re out, you don’t have time to say anything”. When asked if any change had been noted in the way rounds are done, most patients did not notice a change in the process.

##### Issues with Taking Phosphate Binders

A third major theme identified was that patients have multiple issues with taking phosphate binders. Participants indicated a high pill burden and inconsistent schedules made adherence quite challenging. Participants noted that sevelamer tablets are large and “after a while, you set up your diet so it’s like if I’m going to eat at 12… I’m going to take these pills at 11 because it’s like a meal.” One participant indicated it was difficult to remember taking medication when outside of a routine, for instance, while travelling. There was a resounding dislike for TUMS from these participants due to their chalky taste. There was also a general sense of futility noted. Patients discussed a lack of motivation to adhere to medication regimens, in part due to unanswered questions about the timeline of long-term effects of high serum levels of phosphate. For example, one participant said, “They tell you it will do a bit of damage to your veins… but how long… and these are some questions they can’t really answer. Is it going to take ten years? If so, who cares, I’m not going to be here in ten years…”. 

##### Areas for Increased Patient Education

The fourth major theme identified was areas for increased patient education. Participants expressed a desire to have more information regarding renal diet, phosphate binders, abnormal serum levels of calcium, phosphate, parathyroid hormone, and the consequences if these levels are not corrected. They also appreciated the “monthly report card” they receive which has their blood work levels reported and expressed a desire to have this report card given to them more frequently. 

#### 3.2.2. Minor Themes

Four minor themes were identified, including living with chronic illness, satisfaction with care, individualized care, and fluid and dry weight. Minor themes, sub-themes, and representative quotations can be found in Table 6. 

## 4. Discussion

Overall, there were no statistically significant differences in serum markers of CKD-MBD (calcium, phosphorus, PTH) post implementation of the new approach to managing CKD-MBD. There were also no statistically significant differences in medication burden (number of monthly medication changes, number of tablets per day, number of milligrams per day) post implementation of the new approach. From the SAPS survey results, 87% of patients indicated they were satisfied or very satisfied with their care. Patients were generally satisfied with the choices they had in decisions affecting their healthcare (96%) and felt respected by their healthcare professional all (73%) or most of the time (27%). However, when asked about the explanation the healthcare team provides about results of their treatment, 31% of patients indicated they were very dissatisfied. Additionally, 67% of patients surveyed either agreed (48%) or strongly agreed (19%) that the time they had with the doctor or other healthcare professional was too short. From the focus groups, four major themes were identified including lack of privacy, knowledge and perceptions of blood work rounds, issues with taking phosphate binders, and areas identified for increased patient education. Minor themes identified were living with chronic illness, satisfaction with care, need for individualized care, and issues with fluid and dry weight. 

Yorkum et al. conducted a study in Barts and the London NHS trust where a serum phosphate management protocol developed by a dietitian and pharmacist was evaluated over a four-month period and compared with the previous standard of practice. Patients managed using the phosphate management protocol had a significantly greater reduction in serum phosphate levels compared with patients receiving standard practice (−0.22 ± 0.67 vs. +0.19 ± 0.32 mmol/L, *p* = 0.03). It was noted that though statistically significant, this change might not have clinical significance in practice [15]. These results are in contrast to our study that did not find a significant change in phosphate after implementation of the new protocol. With a pharmacist–dietician-led approach to managing CKD-MBD, where a pharmacist was responsible for prescribing related medications under a collaborative practice agreement, there were no differences in serum markers of CKD-MBD or medication burden five months post intervention. This may demonstrate that pharmacists can successfully take on the role of monitoring and prescribing for CKD-MBD with a dietitian as outcomes were similar to traditional nephrologist-led care. 

When surveyed, patients were overall satisfied with the care they received on rounds. However, they indicated dissatisfaction with the explanations the healthcare team provided about their results and with the amount of time they had with the healthcare team on rounds. There was convergence with the survey results and the qualitative results from the focus groups. In the focus groups, patients also expressed the desire to have more detailed information about their blood work and consequences of abnormal results. Additionally, patients mentioned that the time spent with the team on rounds was too short and preferred to have more time allotted. A desire for more information, greater involvement in healthcare decisions, and managing dietary and fluid restrictions has been noted in other qualitative research in this patient population. In a thematic synthesis of the experiences of adults living with hemodialysis, participants wanted more information from their healthcare providers, and some were reluctant to ask questions [7]. Participants also wanted to be listened to and involved in decisions about their care. As with the current study, dietary and fluid restrictions were cited as sources of distress [7]. 

A major strength of this study is its mixed-method design. Focus groups provided qualitative data that supported and enriched the quantitative results. There was convergence between the quantitative and qualitative data, which may strengthen the results. It was also a pre–post design where patients served as their own controls, which limited confounding factors. 

This study has a number of limitations. First, there was a relatively small sample size and the study was largely underpowered. For each analysis, observed power ranged from 1 − β = 0.05 to 1 − β = 0.50. Second, data could only be collected over a nine-month period, which may not have allowed enough data points to detect a true difference. Third, as the study required informed consent, there was an opportunity for volunteer bias, which may have affected the results and reduced the ability for the results to apply to the general population. Fourth, other demographic data, including etiology of CKD, comorbid conditions, dietary intake of phosphate, and dialysate concentrate were not collected. Lastly, with the new approach patients were stratified into three different groups with more conservative or aggressive targets based on various factors. In the previous standard of practice, all patients were treated the same. This may have made it difficult to see a change in serum levels from pre to post intervention.

Many insights gained from this study may be used for future approaches to improve patient care in our center, as well as patient care in similar clinical settings. First, when implementing such a program, it is important to consider areas for increased patient education. This research identified that patients would like increased education on diet, serum biomarkers, and phosphate binders. Second, it is important to consider patient-specific issues related to their treatment. This research identified several patient-specific issues surrounding taking phosphate binders which contributed to poor medication adherence. Optimizing medication regimens for patients and providing helpful information to support adherence to their medications is essential. Lastly, it is paramount to consider ways to improve communication with patients. Lack of privacy was an overarching theme in this study, and patients stated this may impact the amount of information they share with the healthcare team. Ensuring confidentiality during interactions between the patient and healthcare provider should be of upmost importance. Depending on the center, this may require having interactions in a separate room before or after the dialysis session, rather than during the dialysis session.

## 5. Conclusions

The current study demonstrated that a pharmacist–dietician-led, patient-centered approach to managing CKD-MBD did not change serum levels or medication burden in hemodialysis patients after implementation. Identified areas of improvement have been noted and may be used to further develop the process of monthly blood work rounds to ensure they are patient-centered. Through focus groups, areas for increased patient education were identified including renal diet, what serum levels mean and consequences of abnormal serum levels, and phosphate binders. The priority for future research will be to develop a patient education program that addresses knowledge gaps about medication, biomarkers, and diet. Further research is needed to better define patient knowledge gaps and determine how patients would like to receive education. Understanding patient learning styles and preferences will allow the team to develop a formal patient-centered CKD-MBD education program. Additionally, a larger study of longer duration may provide greater evaluation of the novel approach to managing CKD-MBD and may help to continue to improve the process to better suit patient needs.

## Figures and Tables

**Table 1:** Baseline characteristics of participants *.

Characteristic	*n* = 74
Age—yr **(mean, SD)**	67 ± 14.4
Sex—*n* (%)	
Male	44 (59.5)
Female	30 (40.5)
Length of time on dialysis—yr (mean, SD)	4.8 ± 3.2
Transplant status—*n* (%)	
Not a candidate	53 (71.6)
Active	6 (8.1)
Work up	10 (13.5)
On hold	5 (6.8)
Evidence of calcification—*n* (%)	51 (68.9)

* Plus–minus values indicate the mean + standard deviation (SD).

**Table 2:** Serum markers of chronic kidney disease mineral and bone disorder (CKD-MBD).

*n* = 74	Pre Intervention	Post Intervention	*p*-Value	η_p_^2^ *
Calcium—mmol/L (mean, SD) **	2.21 ± 0.17	2.21 ± 0.22	0.983	0.000
Phosphate—mmol/L (mean, SD)	1.73 ± 0.51	1.68 ± 0.69	0.408	0.009
Parathyroid hormone—pmol/L (mean, SD) ***	57.8 ± 33.2	59.1 ± 31.2	0.784	0.001

* ηp2: S = 0.01, M = 0.06, L = 0.14; ** not corrected for albumin; *** intact assay.

**Table 3:** Medication burden.

*n* = 45	Pre Intervention	Post Intervention	*p*-Value	η_p_^2^ *
Number of medications	2.28 ± 1.17	2.23 ± 1.098	0.375	0.143
Number of monthly medication changes	0.32 ± 0.58	0.23 ± 0.484	0.146	0.245
Milligrams/day				
Calcium	2191.11 ±1158.1	2018.9 ± 1125.1	0.054	0.082
Sevelamer	4512 ± 2115.1	4576 ± 2221.4	0.784	0.003
Lanthanum	1687.5 ± 1028.2	1562.5 ± 1161.4	0.391	0.250
Cinacalcet	59.5 ± 29.1	58.6 ± 32.0	0.861	0.001
Cacitriol—oral	0.0004 ± 0.001	0.0003 ± 0.0005	0.400	0.024
Tablets/day				
Calcium	4.16 ± 2.4	3.87 ± 2.3	0.074	0.071
Sevelamer	5.80 ± 2.53	5.88 ± 2.66	0.784	0.003
Lanthanum	2.5 ± 0.577	2.25 ± 0.957	0.391	0.250
Cinacalcet	1.66 ± 0.8	1.68 ± 1.03	0.902	0.001
Cacitriol—oral	0.96 ± 2.17	0.74 ± 1.02	0.319	0.033

* ηp2: S = 0.01, M = 0.06, L = 0.14.

**Table 4:** Responses from the Short Assessment of Patient Satisfaction.

*n* = 48 *	Very Satisfied	Satisfied	Neither	Dissatisfied	Very Dissatisfied
Overall Survey Result (*n* = 48)	(8)	(79)	N/A	(13)	N/A
How satisfied are you with the effect of your treatment/care? (*n* = 52)	37 (71.2)	13 (25.0)	1 (1.9)	1 (1.9)	0 (0)
How satisfied are you with the explanations the doctor/other healthcare professional has given you about the results of your treatment/care? (*n* = 52)	14 (26.9)	15 (28.8)	3 (5.8)	4 (7.7)	16 (30.8)
How satisfied were you with the choices you had in decisions affecting your healthcare? (*n* = 52)	20 (38.5)	30 (57.7)	2 (3.8)	0 (0)	0 (0)
Are you satisfied with the care you received in the hospital/clinic? (*n* = 48)	29 (59.2)	19 (38.8)	1 (2.0)	0 (0)	0 (0)
	**All of the Time**	**Most of the Time**	**Not Sure**	**Some of the Time**	**Never**
How much of the time did you feel respected by the doctor/other healthcare professional? (*n* = 52)	38 (73.1)	12 (26.9)	0 (0)	0 (0)	0 (0)
	**Strongly Agree**	**Agree**	**Not Sure**	**Disagree**	**Strongly Disagree**
The time you had with the doctor/other healthcare professional was too short (*n* = 48)	9 (18.8)	23 (47.9)	6 (12.5)	10 (20.8)	0 (0)
The doctor/other healthcare professional was very careful to check everything when examining you (*n* = 52)	16 (30.8)	31 (59.6)	5 (9.6)	0 (0)	0 (0)

* Note: 48 patients completed the full survey, while 52 patients completed the first 5 questions.

**Table 5:** Major themes and representative quotations.

Major Themes (+ sub Themes)	Quotations
Lack of privacy Withhold information due to feeling uncomfortable in front of others Discomfort with others knowing personal information Dialysis conditions not conducive for privacy Discomfort knowing other patient’s information	“It’s not confidential between doctor and patient because nine times out of ten you’re in a room with somebody else” “No, so you can’t pose a question you might find embarrassing or questionable to somebody else, and you know basically you don’t want anyone knowing your business” “When they’re giving your test results they should be more private”
Knowledge and perceptions of bloodwork rounds Time with healthcare team too short Bloodwork rounds keep you informed Perception of what rounds are and who is involved No change noted in how rounds are done	“You can’t really take the time. Nothing against the doctors but they’re like bang bang bang—you don’t have time to say anything. They’re in, they’re out” “Everything is hey how ya doing, looks good, okay bye”
Issues with taking phosphate binders Sense of futility High pill burden Dislike for TUMS Taking binders outside of routine is challenging	“It’s alright for them to say too much phosphorus, you’re gonna have hardening of your arteries…when? You know 5 years, 2 years, 20 years? If it’s 20 years, why am I getting too excited about it? “The TUMS were too chalky. They weren’t for me” “Well, you’re taking so many pills!”
Areas for increased patient education Renal diet Phosphate binders: how to take and reasons for taking them Monthly “report card” wanted more frequently What levels mean and consequences of abnormal levels	“If you wanna take a binder and keep these papers, you can... at home, you know, scoot back through it and see what’s been up and what’s been down and, you know, almost scrutinize yourself in some of these things. That’s great. But it should be maybe on a monthly basis”

**Table 6:** Minor themes and representative quotations.

Major Themes (+ sub Themes)	Quotations
Living with chronic illness Consequences of dialysis Patients play a role in their own healthcare Dietary modifications	“Well it goes to show really, we’re all in a very delicate situation.” “If you’re getting feedback from the doctor and the results, and you know your potassium levels are high…then maybe you can make some efforts to minimize you intake- you know it’s up to everybody to make sure they’re as health as they want to be.”
Satisfaction with care Feel well monitored A good process Appreciation for the healthcare team Unique and interactive care team	“Personally I feel fairly well monitored… and you know looked after” “It’s actually a very good process”
Individualized care Need to treat people individually Diet is a “touchy balance”	“But I think they gotta start treating people not by the book” “And my point is, we are not a number—we are all individuals”
Fluid and dry weight Concerned about dry weight vs. fluid weight Distrustful of nursing ability to calculate dry weight Dropping dry weight without being informed	“You come in, and they go by your weight, and they deduct the dry weight they set for you… and you turn around and—I’ve seen me come in, go home, and I’m cramping from my toes right up my body because there’s just no fluid in there”

**Figure A1:**
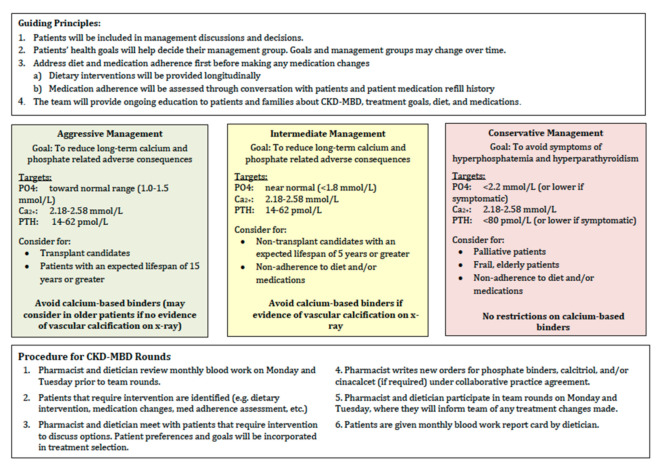
Pharmacist-dietician-led, patient-centered approach to managing CKD-MBD.

## Appendix B

The Short Assessment of Patient Satisfaction

**1. How satisfied are you with the effect of your {treatment/care}?**	
□ *Very satisfied*	0
□ *Satisfied*	1
□ *Neither satisfied nor dissatisfied*	2
□ *Dissatisfied*	3
□ *Very dissatisfied*	4
**2. How satisfied are you with the explanations the {doctor/other health professional} has given you about the results of your {treatment/care}?**	
□ *Very dissatisfied*	0
□ *Dissatisfied*	1
□ *Neither satisfied nor dissatisfied*	2
□ *Satisfied*	3
□ *Very satisfied*	4
**3. The {doctor/other health professional} was very careful to check everything when examining you.**	
□ *Strongly agree*	0
□ *Agree*	1
□ *Not sure*	2
□ *Disagree*	3
□ *Strongly disagree*	4
**4. How satisfied were you with the choices you had in decisions affecting your health care?**	
□ *Very satisfied*	4
□ *Satisfied*	3
□ *Neither satisfied nor dissatisfied*	2
□ *Dissatisfied*	1
□ *Very disssatisfied*	0
□ *Very dissatisfied*	0
□ *Dissatisfied*	1
□ *Neither satisfied nor dissatisfied*	2
□ *Satisfied*	3
□ *Very satisfied*	4
**5. How much of the time did you feel respected by the {doctor/other health professional}?**	
□ *All of the time*	0
□ *Most of the time*	1
□ *About half of the time*	2
□ *Some of the time*	3
□ *None of the time*	4
**6. The time you had with the {doctor/other health professional} was too short.**	
□ *Strongly agree*	0
□ *Agree*	1
□ *Not sure*	2
□ *Disagree*	3
□ *Strongly disagree*	4
**7. Are you satisfied with the care you received in the {hospital/clinic}?**	
□ *Very satisfied*	0
□ *Satisfied*	1
□ *Neither satisfied nor dissatisfied*	2
□ *Dissatisfied*	3
□ *Very dissatisfied*	4

**Scoring**

1. Reverse the scores for items #1, #3, #5, #7.
2. Sum all scores. The score range is from 0 (extremely dissatisfied) to 28 (extremely satisfied).

**Interpreting Scores**

The literature on patient satisfaction shows that between 70% and 90% of patients are satisfied with their health care. This should be kept in mind when interpreting SAPS scores. In general, SAPS scores can be interpreted as follows:

0 to 10 = Very dissatisfied. To obtain a score in this range, a person must have indicated that they are dissatisfied or very dissatisfied on four or more items. Any patient obtaining scores in this range is indicating that their health care has failed them badly and that they are in need of urgent help.

11 to 18 = Dissatisfied. To obtain a score in this range, a person must have indicated that they are dissatisfied or very dissatisfied on at least two items (i.e., two aspects of their health care), or that they have refused to endorse being very satisfied on any item. Patients obtaining scores in this range are indicating health care failure in several areas of their health care and are in need of help in these areas.

19 to 26 = Satisfied. To obtain a score in this range, a person must have indicated that they are very satisfied or satisfied on over half SAPS items (4/7). These patients should be asked about those areas of health care they found unsatisfactory and efforts made to improve such areas.

Hawthorne G, Sansoni J, Hayes L, Marosszeky N, Sansoni E. Measuring patient satisfaction with health care treatment using the Short Assessment of Patient Satisfaction measure delivered superior and robust satisfaction estimates. J Clin Epidemiol. 2014 May;67(5):527-37.

## Appendix C

A Semi-Structured Interview Guide

**Pre and Post:**

- What do you know about the process of monthly blood work rounds?
- Tell me about your experiences with the healthcare team during monthly bloodwork rounds (explain the process of monthly bloodwork rounds to ensure participants know what portion of their healthcare interactions you are referring to)
- Tell me about your level of involvement in the decision-making process when changes are made to your medications?
  ○ How would you like to be included?
  ○ What would that look like for you?
- Tell me about a time you had concerns about your treatment or medications
  ○ Were you given the opportunity to/did you feel you could ask about them?
- What do you think about privacy/confidentiality on the unit?
- Tell me about your experience with taking phosphate binders (these medications include TUMS, calcium, renagel)
  ○ Do you find it difficult to remember taking phosphate binding medication?
  ○ Tell me about any reasons or barriers that stop you from taking your medications.

**Post:**

- Have you noticed a change in your experience in your care?
- Have you felt more involved?

** Note: additional questions may be added around the themes of experience/involvement/engagement/decision making/medications. This is a semi-structured guide for the focus groups.
